# Diagnosis of microbial keratitis using smartphone-captured images; a deep-learning model

**DOI:** 10.1186/s12348-025-00465-x

**Published:** 2025-02-13

**Authors:** Mohammad Soleimani, Albert Y. Cheung, Amir Rahdar, Artak Kirakosyan, Nicholas Tomaras, Isaiah Lee, Margarita De Alba, Mehdi Aminizade, Kosar Esmaili, Natalia Quiroz-Casian, Mohamad Javad Ahmadi, Siamak Yousefi, Kasra Cheraqpour

**Affiliations:** 1 Eye Research Center, Farabi Eye Hospital, Tehran University of Medical Sciences, Qazvin Square, Tehran, 1336616351 Iran; 2 Department of Ophthalmology, University of North Carolina at Chapel Hill, Chapel Hill, NC USA; 3 AI. Health4All Center for Health Equity using ML/AI, College of Medicine, University of Illinois at Chicago, Chicago, IL USA; 4 Virginia Eye Consultants, Norfolk, VA USA; 5 Department of Ophthalmology, University of Tennessee Health Science Center, Memphis, USA; 6 Department of Genetics, Genomics, and Informatics, University of Tennessee Health Science Center, Memphis, USA; 7 Ophthalmological Center After S.V. Malayan, Yerevan, Armenia; 8 University of Illinois College of Medicine, Chicago, IL USA; 9 Department of Cornea and Refractive Surgery, Instituto de Oftalmología, Conde de Valenciana, Mexico City, Mexico; 10 Chashmyar Company, Tehran, Iran

**Keywords:** Keratitis, Microbial keratitis, Artificial intelligence, AI, Deep learning, Diagnosis, Smartphone

## Abstract

**Background:**

Microbial keratitis (MK) poses a substantial threat to vision and is the leading cause of corneal blindness. The outcome of MK is heavily reliant on immediate treatment following an accurate diagnosis. The current diagnostics are often hindered by the difficulties faced in low and middle-income countries where there may be a lack of access to ophthalmic units with clinical experts and standardized investigating equipment. Hence, it is crucial to develop new and expeditious diagnostic approaches. This study explores the application of deep learning (DL) in diagnosing and differentiating subtypes of MK using smartphone-captured images.

**Materials and methods:**

The dataset comprised 889 cases of bacterial keratitis (BK), fungal keratitis (FK), and acanthamoeba keratitis (AK) collected from 2020 to 2023. A convolutional neural network-based model was developed and trained for classification.

**Results:**

The study demonstrates the model’s overall classification accuracy of 83.8%, with specific accuracies for AK, BK, and FK at 81.2%, 82.3%, and 86.6%, respectively, with an AUC of 0.92 for the ROC curves.

**Conclusion:**

The model exhibits practicality, especially with the ease of image acquisition using smartphones, making it applicable in diverse settings.

## Background

Microbial keratitis (MK) typically manifests with a defect in the corneal epithelium that overlays a corneal infiltrate with a response in the anterior chamber. This is accompanied by severe and advancing pain, which may lead to the loss of vision and/or necessitate surgical intervention [1]. MK is the predominant etiology leading to corneal blindness. MK can arise due to a wide array of microbial organisms, including bacteria, fungi, viruses, and parasites. Formerly, it was regarded as a ‘silent epidemic’ in low-income and middle-income nations, exhibiting an annual incidence of 113–799 cases per 100,000 individuals, in comparison to high-income countries with an incidence rate ranging from 2.5 to 4.3 cases per 100,000 individuals annually. MK typically affects individuals during their productive years, thereby accentuating the financial burden experienced by both affected individuals and the nations they reside in [2–4]. In 2010, MK prompted approximately 1 million visits to hospitals or clinical practices in the USA. The cost of treating bacterial keratitis is approximately US$933 per patient [4]. In the UK, hospital admissions for MK have a median cost of £2855, with higher costs for longer admissions and lower socioeconomic status [5]. In Taiwan, outpatient management costs around US$72, while inpatient treatment costs US$1027 [6]. In Australia, severe disease in contact lens wearers costs AU$5500 with an 18-day duration, compared to mild disease costing AU$800 with symptoms lasting 7 days [7]. Disease impact in low-income countries is likely more severe due to limited healthcare access.

Notably, 23–62% of cases experience a decrease in best corrected visual acuity (BCVA) of two or more lines after MK due to corneal scarring, topographical changes, or irregularities due to corneal thinning [8–11]. It is of utmost importance to acknowledge that the result of MK is heavily reliant on immediate treatment following a prompt and accurate diagnosis [12]. The present practice of diagnosing MK by employing slit lamp photography of infectious corneal disease among cornea specialists has been found to yield only 66.0–75.9% accuracy in distinguishing between bacterial and fungal keratitis [13]. The gold standard method for definitively diagnosing MK is corneal scraping and biopsy. The other routine diagnostics include polymerase chain reaction (PCR) and in vivo confocal microscopy. However, these approaches are often hindered by the difficulties faced in low and low middle-income countries where there may be a lack of access to ophthalmic units with clinical experts and standardized investigating equipment, leading to a dependence on empirical treatment and delayed definitive treatment. Additionally, even when microbiological workups are available, the procedure may take several days before yielding any results [14]. These challenges may be held responsible for poorer clinical outcomes and a higher risk of irreversible complications. On the other hand, poorly differentiated clinical features may also contribute to misdiagnosis, which can lead to a disastrous chain of inappropriate treatment, thereby increasing the risk of unidentified clinically essential lesions. This underscores the need for the development of new, more precise, and expeditious diagnostic approaches. These new approaches can be easily accessible in rural areas where other diagnostic options may not be available. Therefore, timely diagnosis can be achieved, and appropriate management will start sooner, ultimately leading to a better prognosis.

Artificial intelligence (AI) has garnered the interest of different medical fields that require visual analysis for making diagnoses. Deep learning (DL), a branch of AI, has shown potential in improving healthcare effectiveness by aiding in automated clinical diagnosis through high-dimensional analysis. Additionally, DL’s availability may reduce the need for costly diagnostic equipment and technicians, enabling better care provision. Moreover, DL could help diagnose eye conditions in areas lacking resources, potentially preventing severe vision loss. Also, the recent COVID-19 pandemic has greatly impacted ophthalmic patients and services, emphasizing the importance of digital health [15, 16]. In ophthalmology, DL has demonstrated diagnostic accuracy comparable to clinical experts in identifying diseases like macular degeneration, glaucoma, and diabetic retinopathy [17–19]. Furthermore, many research groups worldwide, including our team, have assessed the use of DL models in diagnosing various types of MK using corneal photos or confocal images with varying success, thanks to AI advancements [20–25]. A systematic review showed that DL algorithms have great potential in accurately diagnosing and categorizing IK, with performance similar to or possibly even surpassing that of expert corneal professionals [26]. In line with our recent efforts [20, 23, 24], we propose a DL-based model in this study that utilizes photos captured by smartphone cameras to differentiate between important subtypes of MK.

## Materials and methods

### Study design

Participants from Farabi Eye Hospital (Iran), Virginia Eye Consultants (USA), and Nor Hachn Polyclinic (Armenia) were successfully recruited and enrolled in the present study. The inclusion criteria were individuals with a confirmed diagnosis of bacterial keratitis (BK), fungal keratitis (FK), and Acanthamoeba keratitis (AK) between the years 2020 and 2023. The confirmation of the diagnosis was carried out through microbiological culture, in vivo confocal microscopy (IVCM), or PCR. A variety of diagnostic modalities was applied to include confirmed cases as much as possible to enhance the sample size of the study. This approach was similar to the routine clinical practice, in which the diagnosis and proper treatment is made based on one or multi-modality approach. Patients with mixed or other infections, culture-negative cases, individuals with a history of corneal graft procedures such as penetrating keratoplasty, corneal patch grafts, and amniotic membrane grafts, as well as other notable ocular surface conditions that could potentially interfere with the assessments and analyses, were excluded from the study population. Moreover, images exhibiting poor quality, extreme gazes, or incompletely opened eyelids were also excluded, ensuring that only the most valid and representative images were included in the analysis.

**Fig. 1:**
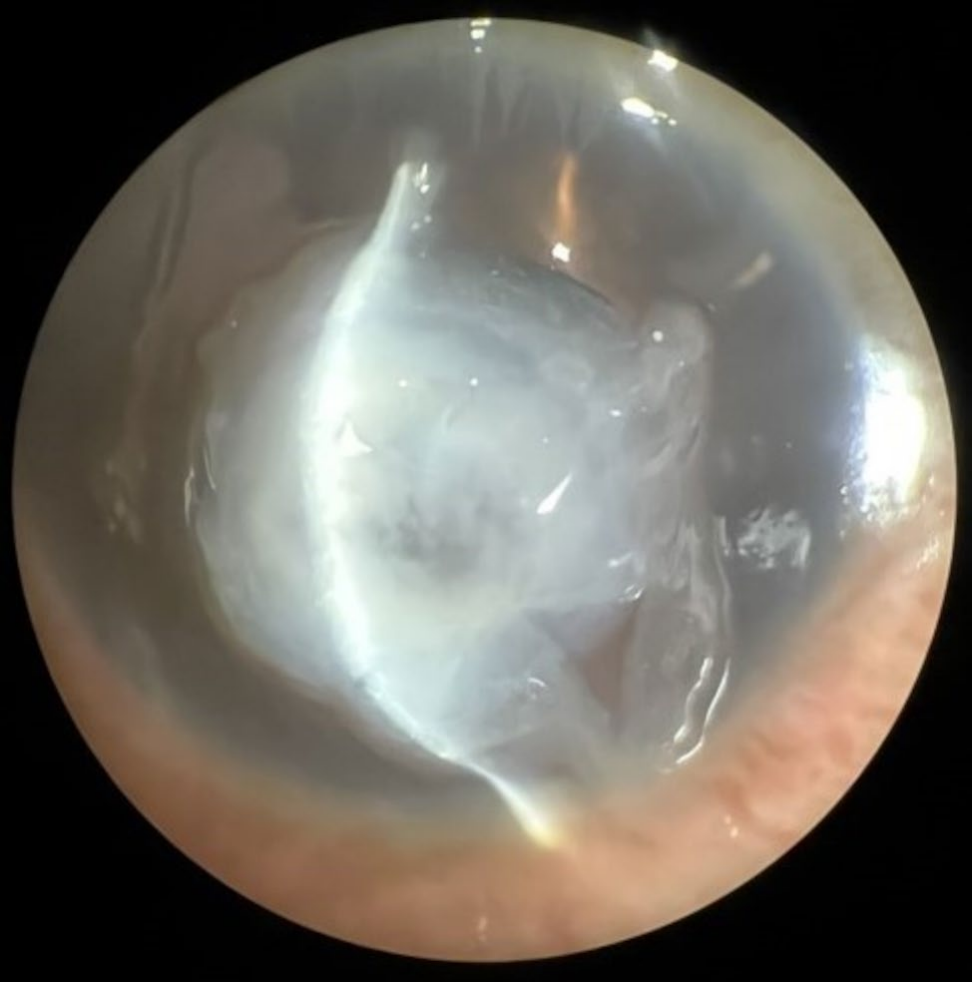
A sample of captured and analyzed image

The images required for the study were meticulously captured using handheld smartphones (iPhone XS and iPhone 13 Pro). To ensure the comfort and safety of the participants, the photography process was carried out following the administration of proper anesthesia, if necessary, which is a standard practice in the field. The participants’ eyelids were deliberately kept open during the photography process, allowing for a comprehensive and accurate examination of the cornea. Additionally, the photography process was conducted under appropriate room lighting conditions, taking into consideration the importance of optimal lighting for capturing clear and informative images. Also, a slit lamp adaptor for smartphones was used to help with consistency and ease of imaging. To capture the photos, 1 to 2 centimeters was established between the camera lens and the eyepiece. The flash was deactivated. The camera was configured to capture images at the highest resolution available. The area of interest was then brought into focus and captured. Deidentified smartphone photos saved as portable network graphics (size: 12–18 MB). To maintain consistency and facilitate the subsequent analysis, all images were cropped, excluding areas beyond the cornea. This cropping process was crucial in removing any irrelevant or distracting elements from the images, thereby enhancing the clarity and focusing on the corneal region, which is of utmost importance in the context of this study (Fig. 1). Furthermore, to ensure comparability and uniformity across all images, a standardization process was implemented, whereby all images were adjusted to a uniform size. This standardization process played a vital role in eliminating any potential bias or confounding factors that may arise from variations in image size, thus ensuring the accuracy and reliability of the subsequent analysis.

As part of the initial data collection phase, a substantial number of external photographs of the eye were meticulously captured and subsequently reviewed by the research team. This extensive data collection process was necessary to ensure that a comprehensive and representative sample was obtained, enabling robust and in-depth analysis. However, in adherence to the rigorous standards set forth by the study protocol, inappropriate images that did not meet the predefined criteria were meticulously excluded from the analysis. After implementing this meticulous exclusion process, a total of 889 high-quality and suitable samples, collected from a diverse group of 98 patients, were included in the subsequent analysis. It is noteworthy to mention that the distribution of these samples across the different diagnostic groups was as follows: 78 images, accounting for approximately 8.77% of the total samples, were collected from the AK group, demonstrating the significance and relevance of this particular subgroup in the overall study. The BK group, on the other hand, contributed the largest proportion of samples, with 479 images, approximately 53.88% of the total. Finally, the FK group comprised 332 photographs, representing approximately 37.34% of the total samples. This distribution highlights the diversity and representation of the sample population, ensuring that the subsequent analysis encompasses a wide range of cases and accurately reflects the characteristics of the target population.

This study adhered to the principles of the Declaration of Helsinki. Ethical clearance was obtained from the Ethics Committee of Farabi Eye Hospital. The requirement for written informed consent was waived by the Ethics Committee. All methods were conducted in compliance with pertinent guidelines and regulations.

### Model design and network details

The computer’s restricted processing power caused the original image resolution to be altered to 300 × 300. Debugging and running the codes were done on a laptop with AMD RYZEN 9 6000 SERIES, 32 GB DDR5 RAM, and Nvidia GEFORCE RTX 3070 Ti with 8 GB VRAM. Python 3.10 was used for developing all codes, while TensorFlow package version 2.12 was utilized for implementing deep learning-based codes on Windows Subsystem for Linux (WSL) version 2.0.

**Fig. 2:**
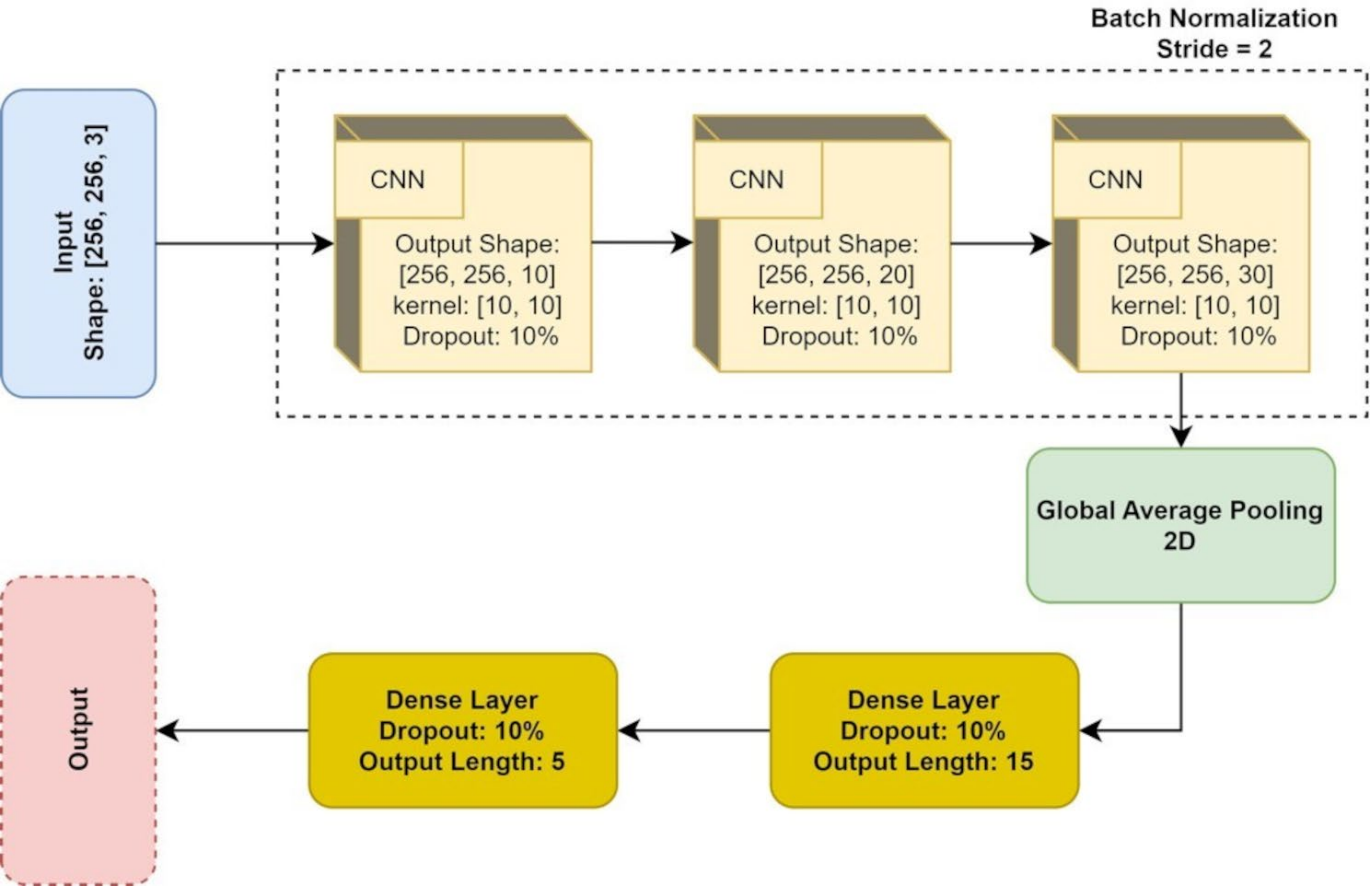
The structure of the designed convolutional neural network (CNN), where the input is an image that may or may not include a diagnosis of infectious keratitis and the output is a value between zero and one indicating the diagnosis of keratitis

In order to achieve our study’s goals, we created a training framework using convolutional neural networks (CNNs), which excel at recognizing image-based tasks. The model was developed to differentiate between different types of MK (AK, BK, FK) using a dataset of 602 smartphone images. The data was divided into 72% for training, 8% for validation, and 20% for evaluation. We utilized a K-fold cross-validation method with K equal to 5. The CNN-based network used in the study is shown in Fig. 2.

Python was used to conduct the simulation, with Tensorflow utilized for deep learning coding. All layers, except the last one, used Relu as the activation function, while Softmax was used for the last layer due to it being a three-class task. The learning algorithm employed was Adam, with a learning rate of 0.001, and cross-entropy served as the loss function. Moreover, both tasks were assigned a batch size of 50 and an epoch number of 200. Class weighting was not necessary as imbalanced classes did not negatively affect the final results.

### Analysis

Sensitivity, specificity, accuracy, precision, F1 score, and R2 score were calculated using Eq. (1) through (6).

1 $$Sensitivity{\text{ }}(\operatorname{Re} call) = \frac{{{\bf{True}}\:{\bf{Positives}}}}{{{\bf{True}}\:{\bf{Positives}} + \:{\bf{False}}\:{\bf{Negatives}}}}$$

2 $$Specificity = \frac{{{\bf{True}}\:{\bf{Negatives}}}}{{{\bf{True}}\:{\bf{Negatives}} + \:{\bf{False}}\:{\bf{positives}}}}$$

3 $$Accuracy = \frac{{{\bf{True}}\:{\bf{Positives}}\: + \:{\bf{True}}\:{\bf{Negatives}}}}{{{\bf{Total}}\:{\bf{population}}}}$$

4 $$Precision = \frac{{{\bf{True}}\:{\bf{Positives}}\:}}{{{\bf{True}}\:{\bf{Positives}}\: + \:{\bf{False}}\:{\bf{positives}}\:}}$$

5 $$F1\,Score = 2 \times \frac{{{\bf{Precision}} \times {\bf{Recall}}}}{{{\bf{Precision}} + {\bf{Recall}}}}$$

6 $$R2\,Score = 1 - \frac{{{\bf{Sum}}\:{\bf{of}}\:{\bf{Squares}}\:{\bf{of}}\:{\bf{Residuals}}}}{{{\bf{Total}}\:{\bf{Sum}}\:{\bf{of}}\:{\bf{Squares}}}}$$

## Results

**Fig. 3:**
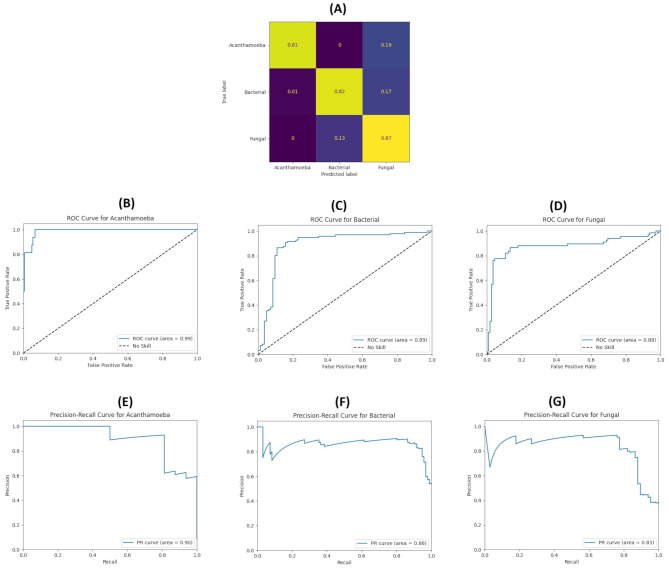
Results of our model. (**A**) Confusion matrix of the model. (**B**) ROC curve of the Acan-thamoeba keratitis group. (**C**) ROC curve of the bacterial keratitis group. (**D**) ROC curve of the fungal keratitis group. (**E**) precision-recall curve of Acanthamoeba keratitis group. (**F**) preci-sion-recall curve of the bacterial keratitis group. (**G**) precision-recall curve of the fungal keratitis group. (For the evaluation phase, 12, 56, and 52 images per fold were used from the classes of Acanthamoeba, Bacterial, and Fungal, respectively. On average, roughly 10, 46, and 45 images, from the classes of Acanthamoeba, Bacterial, and Fungal, respectively, were correctly labeled in the evaluation phase. The presented confusion matrix is based on averaging results of all folds and this estimate is reliable, yet not 100% accurate.)

Table 1 Shows the values of different calculated parameters of our model. Also, Fig. 3 depicts the confusion matrix, ROC curve, and precision-recall (PR) curve of the model for distinguishing each MK subtype. The discrimination accuracy is 0.81 for AK, and for BK and FK, it stands at 0.82 and 0.87, respectively. The ROC curves’ area under the curve (AUC) for AK, BK, and FK are 0.99, 0.89 and 0.88, respectively. The AUC of the PR curve for AK is 0.90, while for BK and FK, it is 0.86 and 0.83, respectively.

**Table 1:** Values of sensitivity, specificity, accuracy, F1 score, and R2 score regarding the model in the evaluation phase

Parameters	Sensitivity	Specificity	Accuracy	Precision	F1 score	R2 score
Performance	83.79%	97.76%	83.80%	84.22%	83.95%	44.50%

## Discussion

Microbial keratitis is an ophthalmic emergency that poses a significant risk to vision and necessitates urgent intervention. The urgency of treatment is heavily dependent on timely diagnosis. Regrettably, the current gold standard diagnostic, corneal scraping and culture, has certain challenges, including low rate of culture positivity and a protracted duration required for culture testing that contributes to a delay in initiating treatment. Notably, deep learning (DL) has demonstrated highly promising results in the diagnosis of MK [26]. In the current study, we have developed a CNN-based model that utilizes DL to effectively distinguish between different subtypes of MK. This model has been specifically designed to leverage the ubiquity and advancements in smartphone technology, allowing for the use of smartphone cameras to capture the necessary photographs for analysis. By utilizing smartphone cameras, this model offers increased portability and cost-effectiveness when compared to traditional slit-lamp imaging setups commonly used for medical image recognition. This is particularly advantageous as it overcomes the limitations associated with slit-lamp imaging and provides easy accessibility to diverse regions, including both developed countries and economically challenged developing regions. Therefore, the current model not only demonstrates a higher level of practicality but also ensures that the benefits of medical image recognition are extended to regions that may not have access to specialized equipment. Our model has been evaluated and has shown an overall discrimination accuracy of 0.838, indicating its effectiveness in correctly classifying different subtypes of MK. Furthermore, when analyzing the discrimination accuracy for specific subtypes, we found that the model achieved an accuracy of 0.81 for AK, 0.82 for BK, and 0.87 for FK, respectively. This comprehensive evaluation of the model’s performance provides further evidence of its potential in the field of computer-aided diagnosis and medical image recognition.

In a multicenter study, Redd et al. similarly constructed a CNN-based model utilizing handheld cameras. The top-performing model was MobileNet, attaining an AUC ranging from 0.83 to 0.86. The CNN ensemble achieved an AUC of 0.84. Their investigation revealed that CNNs exhibited comparatively higher accuracy in identifying fungal ulcers (81%) as opposed to bacterial ulcers (75%) [27]. A similar trend emerged in our results, with the accuracy of FK reaching 88%, slightly surpassing that of BK at 85%. However, it’s noteworthy that the AUCs for both FK and BK were identical, registering at 0.91. The key advantage of the MobileNet model resides in its mobility, making it particularly suitable for integration into telemedicine applications. Notably, our model shares the same potential for versatility in telemedicine settings. To the best of our knowledge, this is the first study introducing a CNN-based model utilizing smartphone photos for the detection of MK. While Redd et al.‘s model utilized photos captured with portable handheld cameras, it was confined to distinguishing between BK and FK [27]. In contrast, our model extends its capability by incorporating the identification of AK. Notably, our model exhibited a higher accuracy in identifying AK compared to both BK and FK.

There are two distinct categories of digital cameras, namely single lens reflex (SLR) cameras and ‘point-and-shoot’ cameras. The selection of either type of camera for usage in medical clinics is contingent upon several factors such as the allocated budget, ease of use, specific photographic requirements, and the proficiency of the user. It is important to note that SLR cameras tend to be heavier, bulkier, and more expensive compared to ‘point-and-shot’ cameras. In addition to these factors, one must also consider the megapixel resolution when making a camera selection. For instance, a 3.2-megapixel camera can adequately fulfill the needs of clinical photography [28]. Nonetheless, it is worth mentioning that smartphones are emerging as a viable alternative to traditional digital cameras. The latest iterations of smartphones boast rear camera resolutions of up to an impressive 50 megapixels, accompanied by image sensors, lens correction capabilities, as well as optical and electronic image stabilization features. Notably, the image quality produced by the smartphone cameras was found to be exceptionally high, on par with the images captured using a slit lamp camera [29]. At present, smartphones are equipped with built-in cameras that are well-suited for slit lamp imaging purposes. The quality of the slit lamp images captured using a smartphone is contingent upon three factors: the resolution of the smartphone camera sensor, the resolution of the slit lamp or microscope, and the focal length of the smartphone camera system.

In a study, Hung et al. employed segmentation models (U2 Net, U-net, and U-net++), with the U2 Net model demonstrating superior performance in cropping slit lamp images [30]. It is plausible that the U2 segmentation model achieved more precise corneal cropping compared to our manual method. Notably, Hung et al. reported that their most effective CNN model achieved an average accuracy of 80.0% in differentiating between FK and BK [30]. Xu et al. employed three classical deep architectures, including VGG16, GoogLeNet-v3, and DenseNet, to develop models for distinguishing subtypes of MK [25]. Their analysis of slit lamp images occurred at three distinct levels: image-level, patch-level, and sequence-level. These levels aimed to capture features from the entire image, the lesion area, and sequences of patch sets, respectively. For patch-level analysis, they utilized manual segmentation, creating patches representing the infectious lesion of the cornea, areas beyond the infectious lesion, conjunctival injection, and anterior chamber exudation. The classification accuracy demonstrated a notable increase, rising from 55.24% at the image-level to 78.73% at the sequence-level. This highlights the improved performance of CNN-based models when attention is focused on the target area of the image, while disregarding irrelevant regions [25]. In our study, we similarly applied manual segmentation to concentrate on the corneas of IK patients, a choice that likely contributed to the high accuracy.

It should be noted that our research included 889 photographs collected from a diverse sample of 98 patients, which implies that some patients may have multiple images represented. The similarity among these photographs could artificially enhance the evaluation metrics. However, we recognized that there were actions we could have undertaken to alleviate these issues as much as possible; the dataset was subjected to multiple shuffles, and we implemented K-fold Cross-Validation while avoiding any augmentation techniques, especially on the evaluation set. Moreover, we took care to manually exclude samples that exhibited a pronounced similarity to other images associated with the same patient. In our dataset, the AK group makes up just 8.77%, while the BK group surpasses over half of the total, which is a notable class imbalance. Despite this issue, the model achieved satisfactory accuracy for each class, and our preliminary experiments did not yield improved results when employing class weighting techniques. Consequently, we opted to present our approach without the application of any class weights.

This study has several limitations. Firstly, the clinical onset of AK typically includes epithelial involvement of the cornea, sometimes manifesting as a pseudodendritic lesion [31]. As the disease advances, it infiltrates the corneal stroma, frequently resulting in a ring-like lesion in the later stages [31]. Consequently, AK can be presented in various scenarios. The limited sample size of 61 images in the AK group may not adequately capture the diverse clinical presentations associated with AK. Secondly, corneal images are recognized for their increased susceptibility to artifacts compared to retinal images. The quality of the photographs can be affected by several factors, such as reflections from the surrounding environment, ambient lighting conditions, and overall image brightness. Despite our efforts to mitigate these artifacts, achieving complete elimination was challenging. Previous studies have shown a significant reduction in the incidence of misclassified data when image brightness was meticulously controlled within a specific range [32]. Also, this study did not include samples from viral keratitis, which is a relatively common presentation in clinical practice. The multi-center design of the study resulted in various photographers capturing the images, potentially affecting the uniformity of the photographs. To address this concern, multiple images were taken of each patient, and only those meeting acceptable quality standards were selected for inclusion. Additionally, while a slit lamp adapter for smartphones was utilized, suggesting that the photographic process may still necessitate more advanced professional equipment, it is noteworthy that the cost of the slit-lamp adapter is significantly lower than that of a camera-mounted slit-lamp. Moreover, transfer learning has the potential to facilitate the development of entirely mobile-based applications in the future.

## Conclusion

In conclusion, this study leveraged deep learning, specifically a CNN, to develop a model capable of distinguishing between different subtypes of MK. Utilizing ubiquitous smartphone cameras, we aimed to enhance portability and cost-effectiveness compared to traditional slit-lamp imaging setups. Our CNN-based model demonstrated practicality in clinical settings, showcasing an overall discrimination accuracy of 0.838. Notably, discrimination accuracy for AK, BK, and FK reached 0.81, 0.82, and 0.87, respectively, with an AUC of 0.92 for the ROC curves. We also described a unique method of manual cropping (to minimize artifact and allow for appropriate analysis) for smartphone images, which are taken through the ocular and have the circular image compared to traditional slit-lamp cameras. Moving forward, continued advancements in deep learning and imaging technologies hold the promise of further refining the accuracy and robustness of models in the field of ophthalmology.

## Data Availability

No datasets were generated or analysed during the current study.

## Abbreviations

MK: Microbial keratitis
BCVA: Best-corrected visual acuity
PCR: Polymerase chain reaction
AI: Artificial intelligence
DL: Deep learning
BK: Bacterial keratitis
FK: Fungal keratitis
AK: Acanthamoeba keratitis
IVCM: In vivo confocal microscopy
CNN: convolutional neural network
ROC: Receiver operating characteristic
AUC: Area under the curve
